# Improved Syringe

**Published:** 1852-10

**Authors:** 


*Improved Syringe.*—Allusion was made, some months since, to an essential improvement in that useful instrument, the syringe. A Boston physician has brought it to a surprising degree of perfection. In workmanship the instruments are unrivalled ; but their real value consists in the applicability of the barrel and flexible tubes to various purposes in maintaining health when mechanical assistance is necessary ; and in fact to every purpose for which a syringe is needed. Both hands of the individual are not required, in operating upon himself, which is a decided improvement. We have not been accustomed to such highly finished articles, in this line, and the country has reason to be proud of them. It is quite needless to particularise their exact construction, to show the superiority of the invention over the common syringes of the shops. Those desirous of an examination are invited to call for that purpose. Facilities for manufacturing are on an extensive scale, such as will at once meet the demand which the utility, compactness, beauty, and economy of the article will create.—*Boston Med. and Surg. Jour.*

